# So Close, yet so Far: Discrepancies between Uveal and Other Melanomas. A Position Paper from UM Cure 2020

**DOI:** 10.3390/cancers11071032

**Published:** 2019-07-22

**Authors:** Manuel Rodrigues, Leanne de Koning, Sarah E. Coupland, Aart G. Jochemsen, Richard Marais, Marc-Henri Stern, André Valente, Raymond Barnhill, Nathalie Cassoux, Andrew Evans, Iain Galloway, Martine J. Jager, Ellen Kapiteijn, Bozena Romanowska-Dixon, Bettina Ryll, Sergio Roman-Roman, Sophie Piperno-Neumann, the UM Cure 2020 Consortium

**Affiliations:** 1 Department of Medical Oncology and INSERM U830, Institut Curie, PSL Research University, 75005 Paris, France; 2 Translational Research Department, Institut Curie, PSL Research University, 75005 Paris, France; 3 Department of Molecular and Clinical Cancer Medicine, University of Liverpool, Liverpool L69 3BX, UK; 4 Department of Cell and Chemical Biology, Leiden University Medical Center, 2333 ZA Leiden, The Netherlands; 5 Molecular Oncology Group, Cancer Research UK Manchester Institute, University of Manchester, Manchester M13 9PL, UK; 6 Department of Genetics, Institut Curie, PSL Research University, 75005 Paris, France; 7 Champalimaud Foundation, 1400-038 Lisbon, Portugal; 8 Department of Translational Research, Institut Curie, PSL Research University, 75005 Paris, France; 9 Department of Ocular Oncology, Institut Curie, PSL Research University, 75005 Paris, France; 10 Melanoma Patient Network Europe, 75597 Uppsala, Sweden; 11 Department of Ophthalmology, Leiden University Medical Center, 2333 ZA Leiden, The Netherlands; 12 Department of Medical Oncology, Leiden University Medical Center, 2333 ZA Leiden, The Netherlands; 13 Department of Ophthalmology and Ocular Oncology, Jagiellonian University Medical College, 31007 Krakow, Poland; 14 Department of Medical Oncology, Institut Curie, PSL Research University, 75005 Paris, France

**Keywords:** uveal melanoma, cutaneous melanoma, mucosal melanoma, conjunctival melanoma, health policy

## Abstract

Despite much progress in our understanding of uveal melanoma (UM) over the past decades, this rare tumour is still often misclassified. Although UM, like other melanomas, is very probably derived from melanocytes, it is drastically different from cutaneous melanoma and most other melanoma subtypes in terms of epidemiology, aetiology, biology and clinical features, including an intriguing metastatic hepatotropism. UM carries distinctive prognostic chromosome alterations, somatic mutations and gene expression profiles, allowing an active tailored surveillance strategy and dedicated adjuvant clinical trials. There is no standard systemic treatment for disseminated UM at present. In contrast to cutaneous melanoma, UMs are not *BRAF*-mutated, thus curtailing the use of B-Raf inhibitors. Although these tumours are characterised by some immune infiltrates, immune checkpoint inhibitors are rarely effective, possibly due to a low mutation burden. UM patients across the world not only face rare cancer-related issues (e.g., specific management strategies, access to information and to expert centres), but also specific UM problems, which can be exacerbated by the common misconception that it is a subtype of cutaneous melanoma. As a European Consortium dedicated to research on UM and awareness on the disease, “UM Cure 2020” participants urge medical oncologists, pharmaceutical companies, and regulatory agencies to acknowledge UM as a melanoma with specific issues, in order to accelerate the development of new therapies for patients.

## 1. Introduction

Uveal melanoma (UM) is a rare intraocular tumour with a stable incidence rate of 5–7 cases per million person-years in populations of European ancestry [1]. UM differs from melanomas of other sites (including conjunctival melanoma, the second most frequent ocular melanoma) in its aetiology, biology, metastatic pattern and treatment (Table 1). Herein, we provide evidence supporting why UM should be considered as a distinct entity and how collaborative actions are undertaken to improve the care of UM patients.

## 2. Biological and Clinical Specificities of UM

While the majority of cutaneous and conjunctival melanomas are associated with ultraviolet (UV) sun exposure and display a subsequent high tumour mutation burden, the typical UV mutational signature has not been identified in UM [2]. Instead, UM shows a remarkably low mutational burden, similar to melanomas arising in the mucosae of the ano, urinary and genital sites [3,4,5]. Cutaneous and conjunctival melanomas are characterised by mutations activating the MAPK (including *BRAF* activating mutations in around half of cutaneous cases), PI3K and receptor tyrosine kinase pathways [6]. A fraction of mucosal melanomas presents with *KIT* mutations that are targetable with KIT inhibitors. In contrast, UMs do not carry targetable *BRAF* or *KIT* mutations, but instead show a distinct somatic mutation profile that is characterised by two main events. The first driver event is an oncogenic activating mutation in either *GNAQ*, *GNA11*, *PLCB4* or *CYSLTR2*, which activates the G_αq_ pathway [4]. The second genetic event consists of either: (i) the bi-allelic inactivation of *BAP1* (~60% of cases), a gene located on chromosome 3p21 that is potentially involved in chromatin organisation; (ii) a change-of-function heterozygous mutation in *SF3B1* (~25% of cases), a subunit of the spliceosome; or (iii) a heterozygous mutation in *EIF1AX* (~15% of cases), a gene involved in the initiation of mRNA translation. These alterations are almost mutually exclusive and currently not targetable.

In contrast to mucosal and acral melanomas, which present with a high number of structural genetic variations [3], UM is characterised by typical chromosomal copy number variation profiles associated with varying prognosis: Monosomy 3 and 8q gain are associated with poor survival while 6p gain predicts a favourable outcome [7,8,9]. These chromosomal aberrations have now been integrated into the list of validated prognostic factors in the 8th edition of AJCC/UICC TNM staging system, along with clinical and pathological features [10]. In contrast, mucosal and acral melanomas present with a high number of structural variations [3]. Interestingly, tumour-infiltrating lymphocytes (TILs) are infrequently observed in primary UM and are associated with chromosome 3 loss, loss of BAP1 expression and poor prognosis [11,12,13]. Cutaneous and conjunctival melanoma typically spread both via the lymphatic as well as the blood system and to a wide variety of organs, such as skin, lung, liver, bone and brain [14,15]. In contrast, UM metastasises haematogeneously, with ~90% of the secondary tumours arising in the liver; liver failure is the main cause of death in the metastatic setting [16].

## 3. Treatment Specificities of UM

The care of primary UM has improved outcomes over the last decades. Nowadays, the primary tumour can be efficiently controlled with brachytherapy, proton beam therapy or surgery. However, up to half of the patients will eventually develop metastases [17]. Therefore, surveillance of these patients is not only focused on the eyes but also on the liver, and may be adjusted for clinical, histological and genomic prognostic factors [8,10].

The hepatotropism observed in UM motivated the development of liver-directed therapies, such as hepatic transarterial chemoembolisation, isolated hepatic perfusion, hepatic intra-arterial chemotherapy, radiofrequency ablation or stereotactic radiotherapy. Prospective and comparative data are limited, but some patients may benefit from these regional therapeutic approaches. Liver surgery remains an interesting therapeutic approach for patients presenting with margin-free resectable disease, with a median overall survival of over two years in a highly selected population of patients [18,19].

While the treatment of metastatic and locally advanced cutaneous melanomas has radically changed in recent years with the emergence of immunotherapy and targeted therapies against B-Raf, Mek and c-Kit activated proteins, no systemic treatment has yet led to any significant improvement in metastatic UM patients’ median overall survival. The latter remains at between 6 to 12 months [16,20]. Cytotoxic agents such as alkylating agents have shown low response rates, similar to cutaneous and mucosal melanomas. Because constitutive activation of the G_αq_ proteins in UM is thought to activate key downstream pathways such as MAPK, protein kinase C and PI3K, inhibition of these pathways has been used in recent clinical trials dedicated to patients with metastatic UM; however, with disappointing results [21,22,23,24,25]. Combination trials are ongoing, based on preclinical investigations (NCT01801358, NCT02273219, NCT02601378).

Immune checkpoint inhibitors, such as CTLA4 and PD-1 inhibitors, are now frequently used as first-line systemic treatment of cutaneous and mucosal melanoma metastases. However, response rates in UM are low, although some outlier responses have recently been reported [13,26,27,28,29,30,31]. Novel immune-based therapies have shown encouraging activity in early phase studies and are currently under investigation (NCT03070392, NCT02363283).

Epigenetic dysregulations are among the most encouraging targets in UM, especially in *BAP1*-mutated tumours, due to the role of BAP1 in chromatin remodelling [32]. Several trials are currently evaluating HDAC inhibitors (NCT02068586, NCT01587352, NCT02697630) and BET inhibitors (NCT02683395), although preliminary results were disappointing. The YAP pathway presents another promising target. Several articles have shown the implication of YAP signalling through a Hippo-independent pathway, with YAP inhibition leading to impressive results in vitro and in animal models [33,34]. Unfortunately, no drugs targeting this pathway are as yet available for patients.

## 4. Future Directions

**Figure 1:**
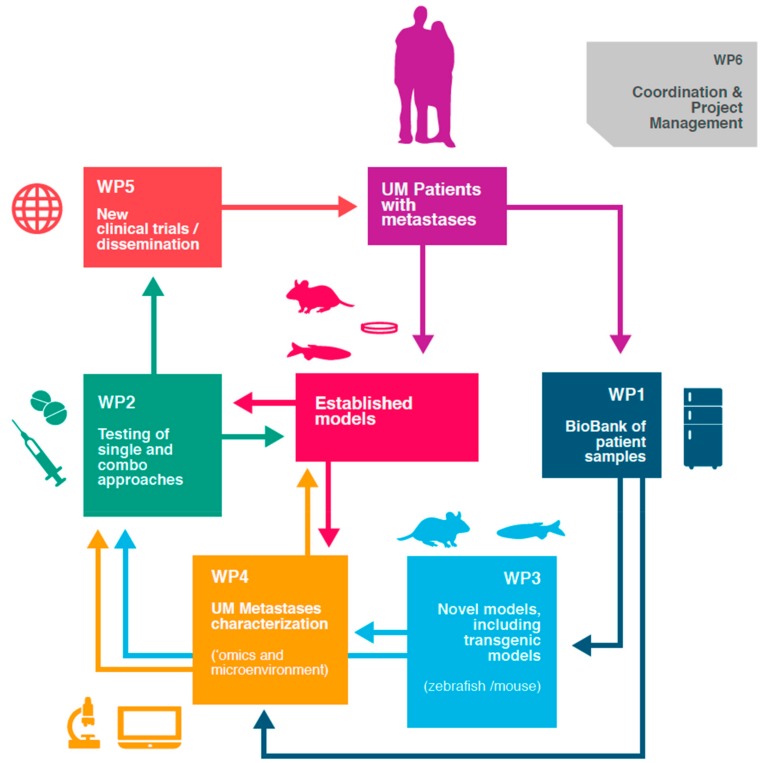
Schematic representation of the UM Cure 2020 work plan. New knowledge of the biology of UM metastases is generated though the characterisation of patient samples (work package (WP1)), the development of genetically modified models (WP3) and several omics approaches (WP4). New therapeutic hypotheses are tested as single agents and combinations (WP2) using relevant preclinical models (WP3), with the objective to translate those findings in the clinic (WP5). Patients are participating in every step of our research, supported by a growing European patient network (WP5).

A few years ago, a group of European researchers, clinicians and patient advocates teamed up in the UM Cure 2020 consortium funded by the European Union Horizon 2020 programme, in order to boost research on metastatic UM (www.umcure2020.org). This 5-year project, focusing mostly on the metastatic disease, aims to reach a better insight into the biology of UM and to test novel treatment modalities with the aim of translating those findings in the clinic (Figure 1). We call upon all of those involved in fundamental, translational and clinical research to urgently join our efforts to improve survival of patients with metastatic UM. The diversity of experimental tools such as large-scale CRISPR/Cas9 synthetic lethality screens, high-throughput drug screening on cell lines, organoids, or patient-derived xenografts for instance now allow researchers to screen for a plethora of new therapies in a cost-efficient, time-limited manner. In addition, we have to improve our understanding of the aetiology and tumorigenesis of UM to have a chance to develop game-changing therapeutic strategies. Research in rare cancers has proven to be efficient to explore biological mechanisms and develop targeted therapies [35,36]. Basic research on UM, for instance, contributed to the description of the downstream G_αq_ pathway and led to the discovery of recurrent splicing abnormalities, resulting in new therapeutic avenues not only in UM but also in other diseases [33,37,38,39]. Understanding the mechanisms implicated in drug resistance and in exceptional responders could also provide innovative biomarkers and directions for new treatments [31,40,41]. To reach this goal, future UM trials should anticipate biological analyses by collecting samples and sharing the resulting data.

However, available slots for UM patients in current early phase trials are limited. This is becoming more evident in the current immunotherapy revolution. Next-generation immunotherapy trials preferentially include patients with immunotherapy-sensitive tumour types (e.g., cutaneous melanomas, lung carcinomas or hypermutated tumours), and thus limit places for patients with metastatic UM. There is an absolute urgency to open recruitment slots for UM patients with advanced disease as these new trials often combine different agents to increase immunogenicity of tumours, and thereby will possibly unleash an immune response to metastatic UM, which are otherwise considered ‘cold’ tumours. Basic research on the specific immunological behaviour of UM is particularly needed in our search for effective therapies.

Development of new drugs dedicated to patients with UM is crucial, but the numerous setbacks made this field harsh and unattractive for pharmaceutical companies. However, the real possibility of an ‘orphan drug designation’ is a strong incentive for them to invest in this field. UM is a rare tumour but large randomised phase III trials are feasible as proven before with the SUMIT and the EORTC 18021 trials in the metastatic setting, and the FOTEADJ trial in the adjuvant setting (NCT01974752, NCT00110123, NCT02843386). Support from international clinical networks of UM experts, such as the European Network for Rare Adult solid Cancers (EURACAN) and the International Rare Cancers Initiative (IRCI) can help to develop large UM trials.

In parallel to strengthening research efforts towards uncovering new treatments to be tested in the clinic, one of the first pragmatic actions that must be implemented is to increase the importance of palliative care. Indeed, palliative care has been one of the major developments in oncology in the last decades. Prevention and early treatment of the symptoms of metastatic disease improves quality of life and, according to some studies, may impact overall survival [42]. Supporting such care actions and developing research in this field might have quick and direct consequences on the quality of life of patients and caretakers while reducing the costs of unnecessary hospitalisations and improper treatments.

Last but not least, the role of patient advocacy groups should be reinforced. Patient advocacy groups not only operate as patient guides; they are acquiring deep systems knowledge into the drug research and development process and are now involved in research projects, scientific advisory boards, clinical trial steering committees and as reviewers of scientific publications. Authorities such as the European Medicines Agency and national Health Technology Assessment bodies have been increasing their interaction with patient groups. Rare cancers are, paradoxically, a common problem. They account for a fifth of cancer diagnoses, and still each entity presents with very specific biological features and therapeutic needs, which makes a global understanding of all these tumour types unrealistic. Such awareness should encourage health authorities to discuss current evidence with UM experts when evaluating UM-related questions. Collaboration between researchers, doctors, patients and families alongside with health authorities is vital, in order to improve the outcome of this grim disease and finally offer some hope to patients suffering from UM.

## 5. Conclusions

UM is a melanoma with distinct biological and clinical features that differs significantly from cutaneous and other melanoma subtypes. Specific efforts are required to accelerate fundamental and clinical research, expand access to clinical trials and raise awareness about this disease. UM Cure 2020 is a unique consortium of patients, preclinical, translational and clinical European experts in UM, to specifically tackle the challenge of metastatic UM. Our objective is to develop improved treatment strategies for the metastatic setting through a tight collaboration between different stakeholders, with the ultimate aim to initiate UM-dedicated clinical trials.

## Figures and Tables

**Table 1:** Main clinical and biological features of melanomas.

Main Features	Cutaneous Melanoma	Uveal Melanomas	Mucosal Melanomas	Conjunctival Melanomas
**Relative Incidence**	~90% of melanomas	~5% of melanomas	~2% of melanomas	~1% of melanomas
**Etiological Factors**	Sun, melanocytic nevi, heredity in ~2% of cases (mostly *CDKN2A*)	Mostly unknown, Ota nevi, heredity in ~1% of cases (*BAP1*)	Unknown	Sun, primary acquired melanosis, conjunctival melanocytic nevi
**Clinical Presentation**	Satellite and in transit, lymph node and visceral metastases	Mostly liver metastases	Visceral metastases	Satellite and in transit, lymph node and visceral metastases
**Mutation Burden**	High	Very low	Low	High
**Recurrent Mutations**	*BRAF*, *NRAS*, *KIT*, *NF1*, *CDKN2A*	*GNAQ*, *GNA11*, *PLCB4*, *SF3B1*, *BAP1*, *SF3B1*, *EIF1AX*	*KIT*, *NRAS*, rare *BRAF*	*BRAF*, *NRAS*, *KIT*, *NF1*
**Therapeutic Options**	PD1 inhibitors, BRAF/MEK inhibitors	PD1 inhibitors, chemotherapy	PD1 inhibitors, KIT inhibitors (not authorised)	PD1 inhibitors, BRAF/MEK inhibitors
